# Intraoperative navigation system with a multi-modality fusion of 3D virtual model and laparoscopic real-time images in laparoscopic pancreatic surgery: a preclinical study

**DOI:** 10.1186/s12893-022-01585-0

**Published:** 2022-04-11

**Authors:** Chengxu Du, Jiaxuan Li, Bin Zhang, Wenfeng Feng, Tengfei Zhang, Dongrui Li

**Affiliations:** 1 Department of Hepatobiliary Surgery, The Second Hospital of Hebei Medical University, 215 Heping West Road, Hebei 050000 Shijiazhuang, China; 2 Hebei Medical University, Shijiazhuang, Hebei China; 3 School of Artificial Intelligence, Beijing University of Posts and Telecommunications, Beijing, China; 4 Department of Medical Imaging, The Second Hospital of Hebei Medical University, Hebei Shijiazhuang, China

**Keywords:** Navigation, Laparoscopy, 3D virtual model, Image fusion, Pancreatic surgery

## Abstract

**Background:**

Laparoscopy is widely used in pancreatic surgeries nowadays. The efficient and correct judgment of the location of the anatomical structures is crucial for a safe laparoscopic pancreatic surgery. The technologies of 3-dimensional(3D) virtual model and image fusion are widely used for preoperative planning and intraoperative navigation in the medical field, but not in laparoscopic pancreatic surgery up to now. We aimed to develop an intraoperative navigation system with an accurate multi-modality fusion of 3D virtual model and laparoscopic real-time images for laparoscopic pancreatic surgery.

**Methods:**

The software for the navigation system was developed ad hoc. The preclinical study included tests with the laparoscopic simulator and pilot cases. The 3D virtual models were built using preoperative Computed Tomography (CT) Digital Imaging and Communications in Medicine (DICOM) data. Manual and automatic real-time image fusions were tested. The practicality of the navigation system was evaluated by the operators using the National Aeronautics and Space Administration-Task Load Index (NASA-TLX) method.

**Results:**

The 3D virtual models were successfully built using the navigation system. The 3D model was correctly fused with the real-time laparoscopic images both manually and automatically optical orientation in the preclinical tests. The statistical comparative tests showed no statistically significant differences between the scores of the rigid model and those of the phantom model(*P* > 0.05). There was statistically significant difference between the total scores of automatic fusion function and those of manual fusion function (*P* = 0.026). In pilot cases, the 3D model was correctly fused with the real-time laparoscopic images manually. The Intraoperative navigation system was easy to use. The automatic fusion function brought more convenience to the user.

**Conclusions:**

The intraoperative navigation system applied in laparoscopic pancreatic surgery clearly and correctly showed the covered anatomical structures. It has the potentiality of helping achieve a more safe and efficient laparoscopic pancreatic surgery.

## Background

Laparoscopy is widely used in pancreatic surgeries nowadays [1–3]. Laparoscopic pancreatic surgeries are complex procedures associated with high morbidity due to the involvement of intricate organs and major vascular structures. The efficient and correct judgment of the anatomical structures is crucial for a safe laparoscopic pancreatic surgery. The technology of 3D visualizing model of the organs and major vascular structures based on the imaging DICOM data and multi-modality imaging fusion is realized by the segmentation and registration algorithms using the computer programming language [4, 5]。The technology has already been clinically applied in neurosurgery [6–9] and orthopedics [10–13]. Reports on the use of the technology in preoperative planning and intraoperative navigation in hepatectomy and nephrectomy exist [14–19]. However, there is no report of its use in the navigation system for laparoscopic pancreatic surgery. In the current study, we developed an intraoperative navigation system for laparoscopic pancreatic surgery using manual and automatic optical orientation image fusion of the preoperative 3D model and real-time laparoscopic images to observe the tumor inside the pancreas and peripheral blood vessels. We also tested the accuracy and evaluated the practicality of this navigation system both in preclinical tests and pilot clinical cases.

## Methods

### Intraoperative navigation system

#### Hardware

The hardware included a computer with the intraoperative navigation system, a monitor screen with a video-exporting cable connected to the laparoscopic screen, and an automatic optical orientation system (POLARIS Vicra, Northern Digital Inc., Waterloo, Canada), including an infrared emission device and reflection markers.

#### Software

The software for the navigation system was developed ad hoc. The functions of the intraoperative navigation system included 3D virtual modeling and image fusion.

3D virtual modeling was realized by importing the CT DICOM data of the subjects into the intraoperative navigation system. The 3D virtual model of the patients included the tumor, the organs and peripheral blood vessels, which were built based on the DICOM data of the preoperative enhanced thin-slice CT scan. The machine learning algorithms included the Fisher Linear Discriminant and Graph-cut Algorithms.

Image fusion was realized manually or using the automatic optical orientation function. While designing the system, we ensured that the 3D model could be superimposed onto the real laparoscopic image. Using the manual image fusion, the orientation and size of the 3D model were adjusted by the operator for a more accurate superimposition with the laparoscopic image. Using the automatic-optical-orientation image fusion, the orientation of the 3D model displayed on the fusion image was determined by the optical orientation system, and the size of the 3D model was adjusted by the operator. During the tests, the optical markers were fixed to the subject and the handheld part of the laparoscope. The position of the optical markers fixed on the subject during the tests had to be the same as that during the CT scanning. The optical orientation system could track both the movement of the laparoscope and the site of the subject to obtain a merged laparoscopic view with the 3D image of the anatomical structures automatically.

### Operators of the navigation system

A total of 10 surgeons with different levels of laparoscopic pancreatic surgical experience, including 2 Interns, 2 Residents, 2 Fellows, 2 Attendants and 2 expert surgeons, participated in the preclinical study. The two expert surgeons also operated on one each of the two clinical pilot cases.

### Subjects of the preclinical tests

**Fig. 1:**
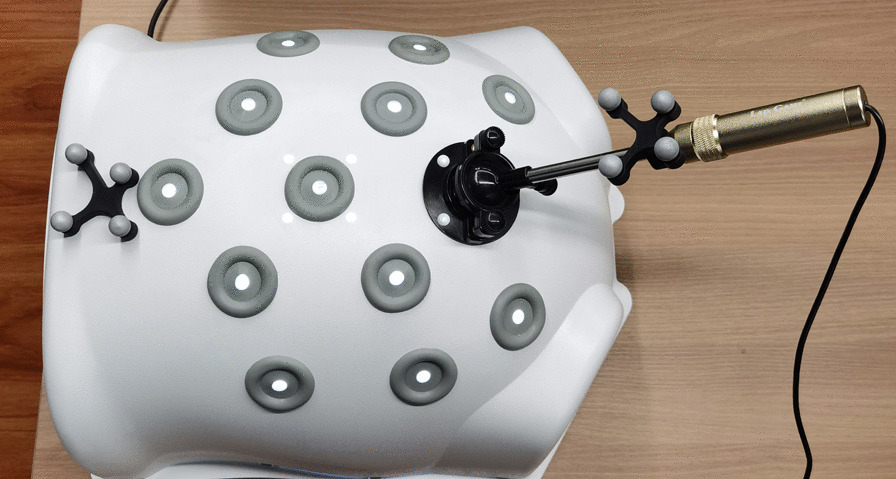
Laparoscopic simulator with rigid or phantom models fixed inside and optical markers fixed on the outer case and the laparoscopy

A laparoscopic simulator (Lap Game, BellySim, Hangzhou Jingyou Technology Inc. Hangzhou, China) with rigid or phantom models fixed inside was used for the preclinical tests. The rigid models, which had no shape deformation, were used to initially test the accuracy of the image fusion. The phantom models, which had shape deformation to mimic the abdominal organs, were used to test the accuracy of the image fusion in soft tissues. To better mimic the complete or partial covering of the tumor, the organ and the blood vessels by other organs or tissues, we designed the corresponding models. The covers could be removed with the laparoscopic instruments during the tests. The optical markers of the optical orientation system were fixed on the laparoscope and the outer case of the laparoscopy simulator (Fig. 1).

### Two pilot clinical cases

The two pilot cases included a laparoscopic pancreaticoduodenectomy case and a laparoscopic pancreatosplenectomy case The two expert surgeons performed the surgeries, one each, of the two cases.

Case 1: A 68-years-old female admitted for jaundice was diagnosed with a periampullary tumor. After percutaneous transhepatic cholangial drainage, a laparoscopic pancreaticoduodenectomy was performed. The pathological diagnosis was common bile duct adenocarcinoma.

Case 2: A 40-year-old female was admitted after diagnosis of the pancreatic body and tail tumor. A laparoscopic pancreatosplenectomy was performed. The pathological diagnosis was a pancreatic solid pseudopapillary tumor.

## Preclinical tests

The 3D modeling was performed as follows: (1) With optical markers fixed on the outer case and rigid or phantom models fixed inside, the laparoscopic simulator had a preoperative thin-slice CT scan. (2) The CT DICOM data was imported into the navigation system. (3) The operator selected the area of interest using the CT images. 4.The 3D virtual model, including all the anatomical structures that the operator wanted to see, was developed. If the 3D model was unsatisfying, step 3 was redone. After constructing the 3D models, the operators recorded the assessments of the 3D models (satisfactory or non-satisfactory).

During laparoscopy, image fusion was performed. First, the operators exported the laparoscopic real-time video to the navigation system to display it on the navigation system screen. Then, the operators ran the fusion function to construct the 3D model as shown on the screen overlapping the real-time images. Then, the operators ran the manual or automatic fusion function. The site, orientation, size, and transparency of the 3D model could be manually adjusted. If the automatic fusion function was chosen, the 3D model would overlap with the real-time images at a certain site with a certain orientation determined using optical tracking, while the size and transparency of the 3D model had to be adjusted manually. If the manual fusion function was chosen, the site, orientation, size and transparency of the 3D model were manually adjusted to obtain the best overlap. Finally, when the operators were satisfied with the image fusion, they removed the covering with the laparoscopic instruments to reveal the covered structures to evaluate the accuracy of the image fusion. Both fusion functions were tested in the preclinical tests.

### Evaluation of the accuracy and the practicality

The preclinical tests included 4 parts, which were a rigid model with automatic fusion, a rigid model with manual fusion, a phantom model with automatic fusion and a phantom model with manual fusion. The 10 participants recorded their evaluations on the accuracy of image fusion (good, average, or bad). Therefore, a total of 40 evaluations were collected and analyzed. The NASA-TLX workload measurement [20] was applied to evaluate the practicality of the intraoperative navigation system. In the NASA-TLX workload measurement, a score of 1 indicated very low while that of 20 indicated very high mental or physical demand, time consumption, effort, or frustration of the participants while using the navigation system (Table 1). Therefore, an aggregate score of 6 indicated for success of the navigation system while 120 indicated failure. All the participants used the intraoperative navigation system and completed the NASA-TLX workload measurement in each test. The scores of all the tests were recorded and statistically analyzed.

**Table 1:**
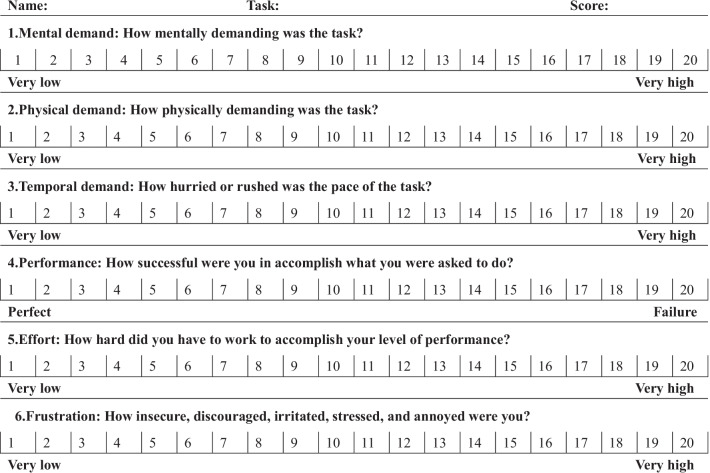
The NASA-TLX workload measurement

### Pilot clinical cases

Case 1 had a laparoscopic pancreaticoduodenectomy for distal cholangiocarcinoma. Case 2 had a laparoscopic pancreatosplenectomy for pancreatic body and tail tumor. The enhanced thin-slice CT scans were obtained in both cases. The CT DICOM data were imported into the navigation system to build the 3D model using the method described in the preclinical tests before the surgery. During the surgeries, only the manual fusion function was used to avoid the possible interference of the optical navigation system in the surgeries and injury to the patients from the parts of the optical navigation. The fusion function was run with the method described in the preclinical tests. The intraoperative data, including operation time, blood loss and transfusion volume, and short outcome of the two cases were recorded and analyzed. The operators recorded the evaluation results of the image fusion and completed the NASA-TLX workload measurement. The study was performed in accordance with the relevant guidelines and regulations and was approved by the Research Ethics Committee of the Second Hospital of Hebei Medical University, Shijiazhuang, China. Informed consent was obtained from all the study subjects.

### Statistical analyses

The variables of the NASA-TLX workload measurement were presented as median with IQR and compared using Mann-Whitney *U* tests. The statistical analyses were conducted using IBM SPSS Statistical 26.0 (IBM Corp, Armonk, NY). A *P*-value of less than 0.05 was the criterion for statistical significance.

## Results

### Preclinical tests

**Fig. 2:**
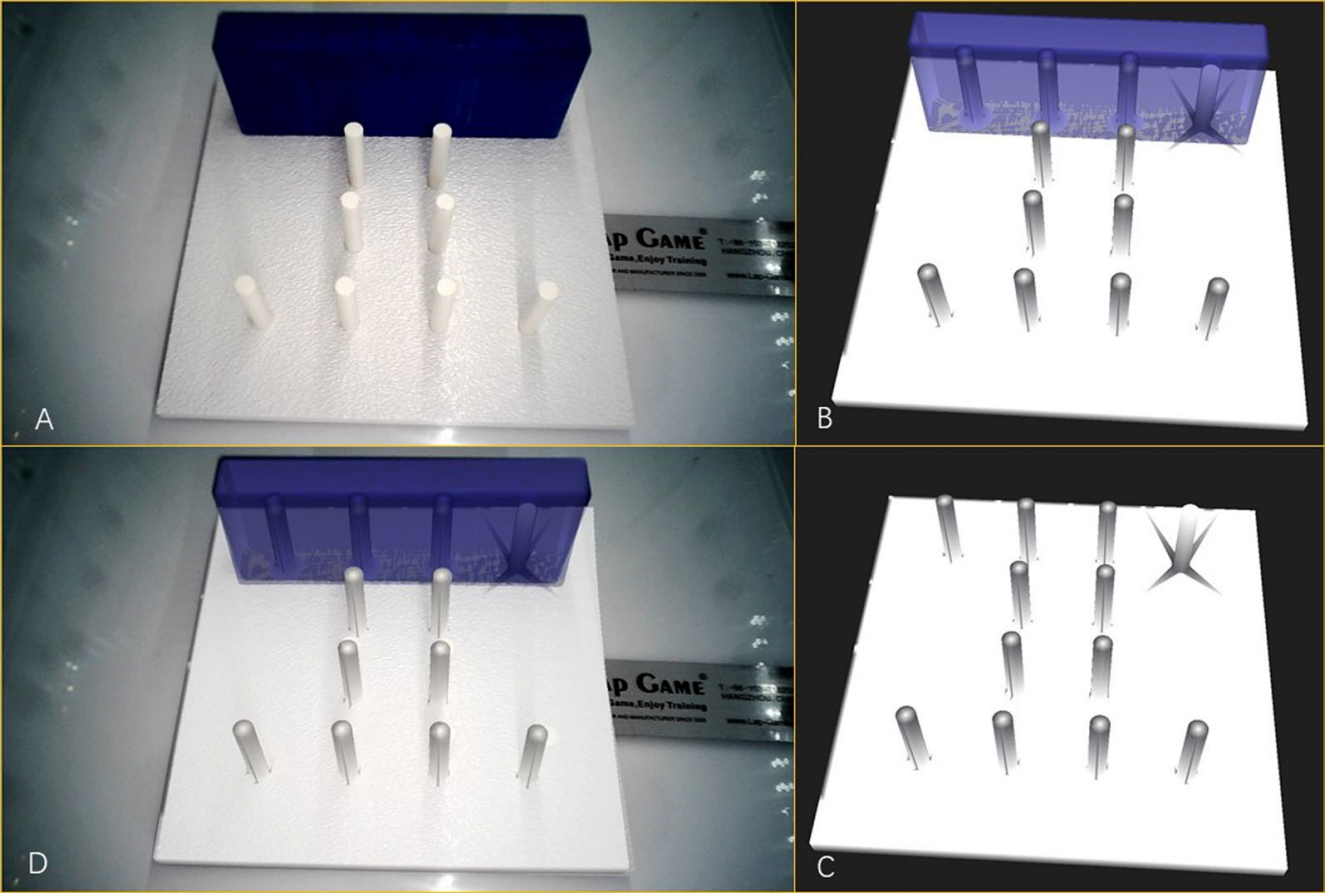
**A** The laparoscopic visual field of the simulator. **B** The 3D model of the rigid model with a covering. **C** The 3D model of the rigid model without a covering, showing the structures inside. **D** The fusion image of 3D model and laparoscopic visual field, showing the covered structures

**Fig. 3:**
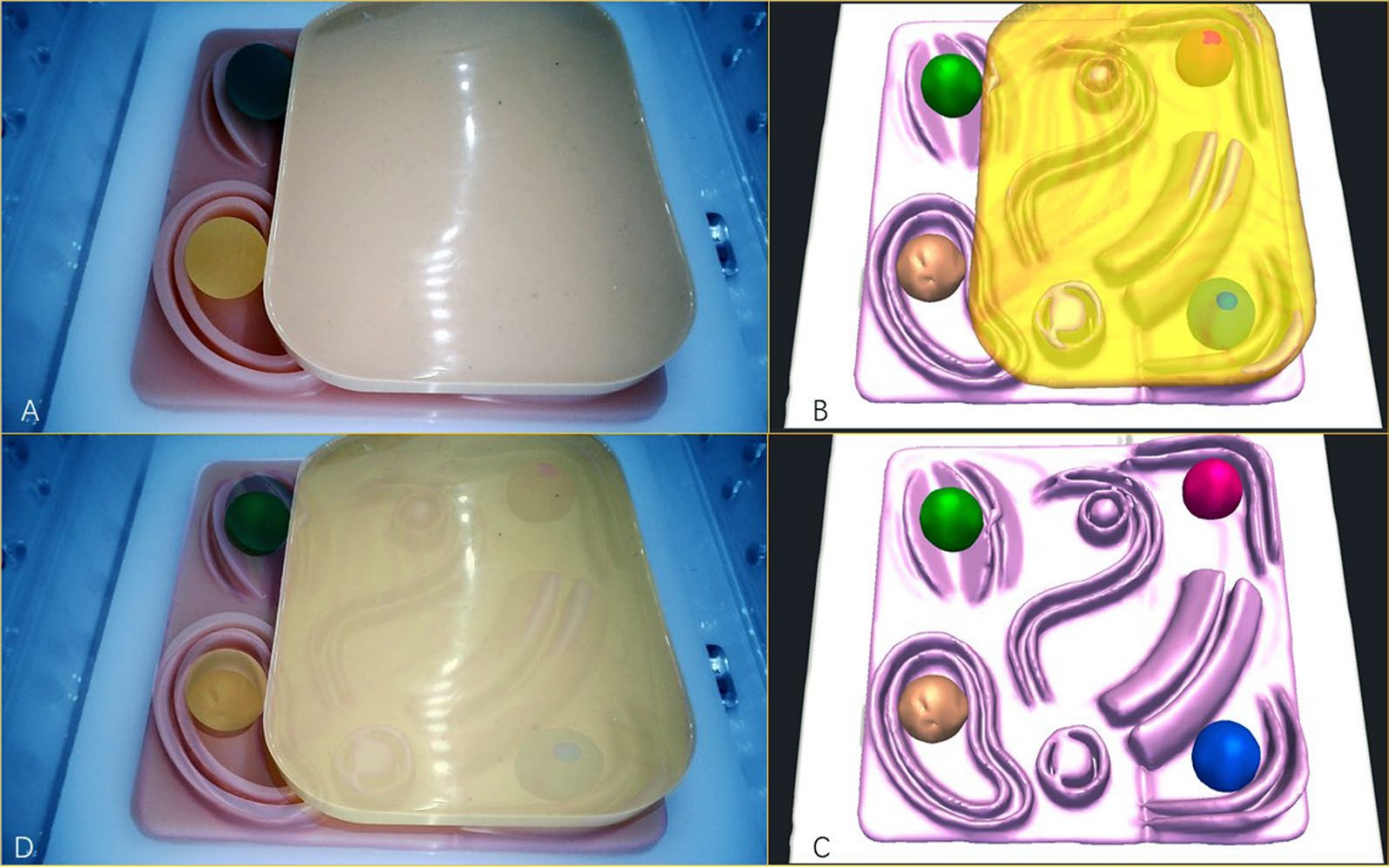
**A** The laparoscopic visual field of the simulator. **B** The 3D model of the phantom model with a covering. **C** The 3D model of the rigid model without a covering, showing the structures inside. **D** The fusion image of 3D model and laparoscopic visual field, showing the covered structures

3D virtual models were successfully built. The satisfaction rate was 100%. Accurate real-time fusion images were achieved by both manual adjustment and automatic optical orientation (Figs. 1 , 2 and 3). The evaluations on the accuracy of image fusion in all the tests were “Good”. The practicality of the intraoperative navigation system was evaluated by the operators using NASA-TLX method. The medians and the interquartile ranges of the scores for mental demand, physical demand, temporal demand, performance, effort, and frustration are shown in Table 2. The medians of the total score were 28 for the rigid model with manual fusion, 31 for phantom model with manual fusion, 25 for the rigid model with automatic fusion and 27 for the phantom model with automatic fusion. The statistical comparative tests showed no statistically significant differences between the scores of the rigid model and those of the phantom model(*P* > 0.05). There was statistically significant difference between the total scores of automatic fusion function and those of manual fusion function (*P* = 0.026) (Table 2). The results suggested that the intraoperative navigation system worked well and was easy to use for the operator. The automatic fusion function brought more convenience to the user.

**Table 2:** Scores of the NASA-TLX workload measurement (median and interquartile ranges of the parameter). [ M (Q1, Q3)]

Variable	Manual			Automatic			*P*
	Rigid	Phantom	*P*	Rigid	Phantom	*P*	
Mental demand	5 (3.25,5.75)	6 (4.5,7.75)	0.218	4 (3,6.5)	5 (3.25,9)	0.315	–
Physical demand	7 (6,7)	8 (5.25,8.75)	0.280	5 (4,5.75)	5 (4,5)	0.971	–
Temporal demand	5 (4.25,6.75)	6 (4,7)	1.000	3 (2.25,5.75)	5 (4,5)	0.393	–
Performance	2 (2,3)	4 (2,5)	0.063	3 (2.25,5)	4 (3,6.75)	0.393	–
Effort	3 (2.25,4)	5 (3,6.75)	0.063	2 (2,4.75)	3 (2,5.75)	0.436	–
Frustration	1 (1,2.5)	1 (1,2.75)	0.631	1 (1,4.5)	1 (1,4.5)	1.000	–
Total	28 (24.25,38.75)	31 (25.75,36)	0.971	25 (19.75,25)	27 (25,28.75)	0.105	–
	29 (24.75,36)		–	25 (21.5,28)		–	0.026

### Clinical outcome of the pilot cases

**Fig. 4:**
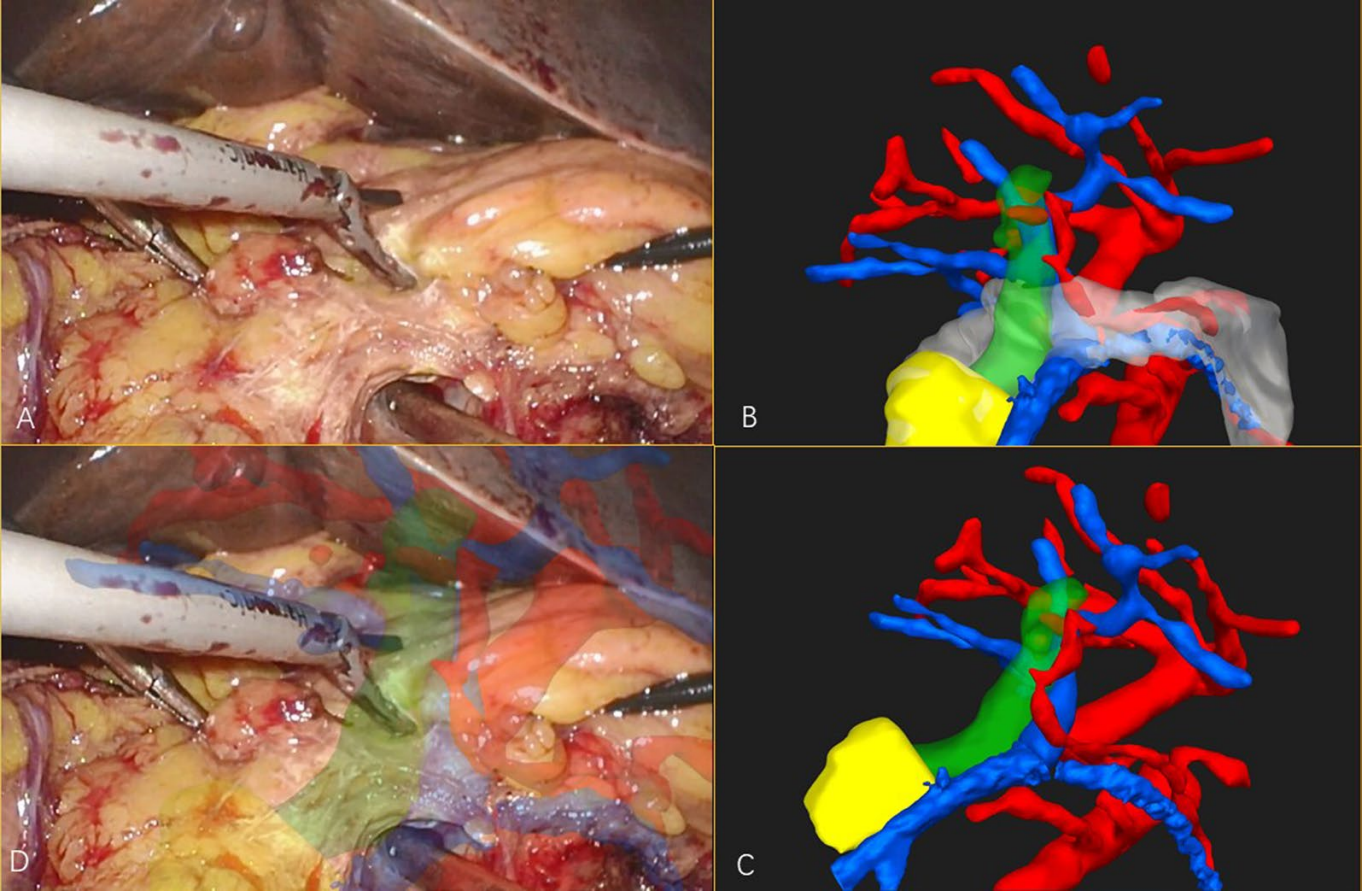
**A** The laparoscopic visual field of case 1. **B**, **C** 3D model of pancreas, tumor and vascular structures. **D** The fusion image of 3D model and laparoscopic visual field, showing the tumor and vascular structures in deep tissue

**Fig. 5:**
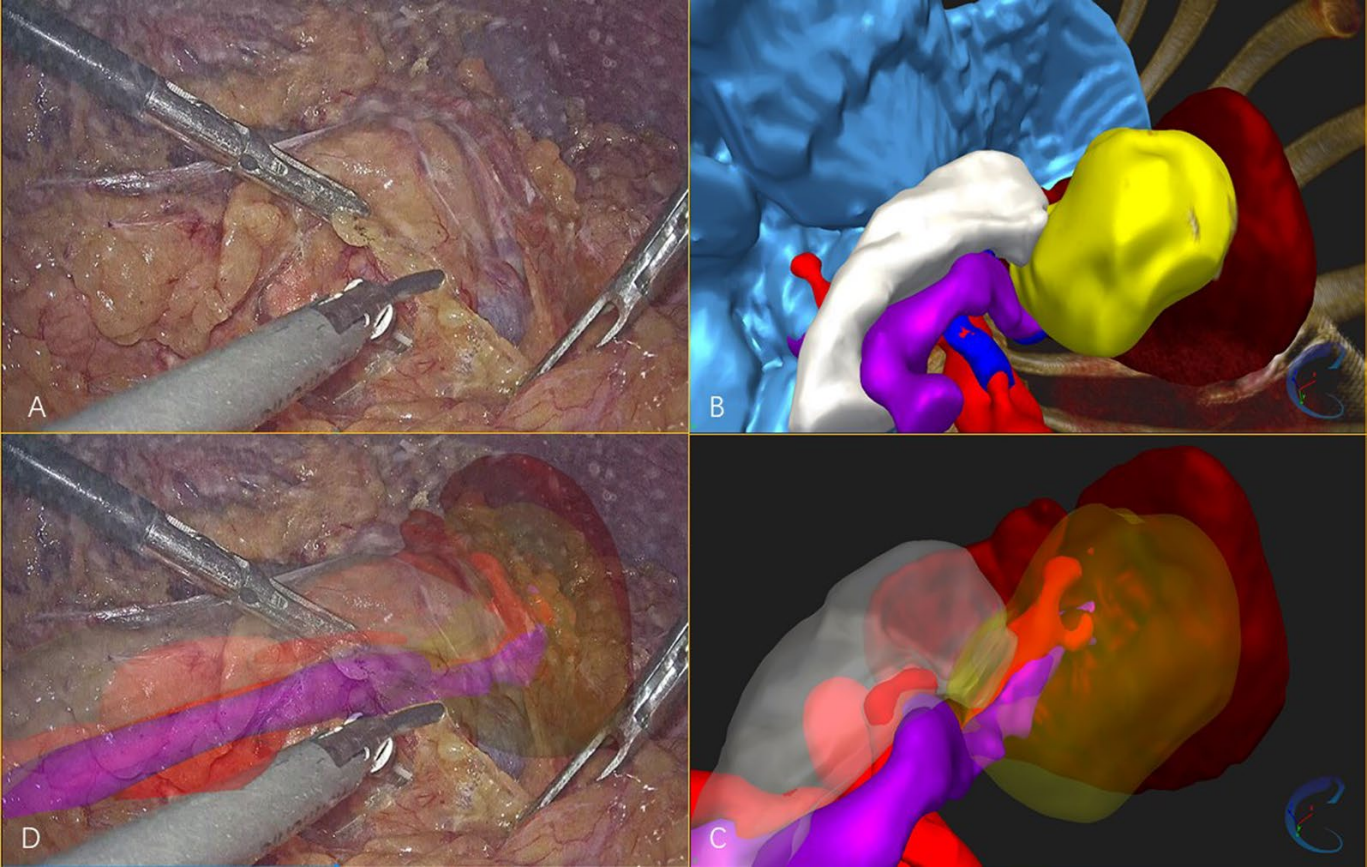
**A** The laparoscopic visual field of case 2. **B**, **C** 3D model of organs, tumor and main blood vessels. **D** The fusion image of 3D model and laparoscopic visual field, showing the tumor and main blood vessels in deep tissue

In the pilot cases, 3D models were successfully built and accurate real-time fusion images were achieved by manual adjustment (Fig. 4 for Case 1, and Fig. 5 for Case 2).

Case 1: The laparoscopic pancreaticoduodenectomy was completed with an operation time of 270 min. The intraoperative blood loss was 300 mL and no transfusion was required. The patient recovered well with no post-operative complications and was discharged on the 10th day after the surgery.

Case 2: The laparoscopic pancreatosplenectomy was completed with an operation time was 180 min. The intraoperative blood loss was 100 mL and no transfusion was required. The patient recovered well with no post-operative complications and was discharged on the 5th day after the surgery.

## Discussion

Laparoscopic pancreatic surgery is a minimally invasive surgery with a clear visual field and delicate surgical manipulation [21]. It is reported that the clinical outcomes of laparoscopic surgery are as good as, or even better than those of open surgery [22, 23]. However, a surgeon needs to take a lot of time and effort to achieve the proficiency in laparoscopic pancreatic surgery [24, 25]. Dissection of the main blood vessels is a key step for pancreatic surgery [26]. In the current study, we tried to use advanced technology to provide an effective method to accurately locate the main blood vessels to shorten the operation time, reduce the degree of technical difficulty and accelerate the learning curve of laparoscopic pancreatic surgery.

The technologies of 3D virtual model and image fusion are widely used in the medical field for preoperative planning and intraoperative navigation [4–19]. General surgeons mainly apply the technology for hepatectomy, dealing with the problems of deformation and displacement [14]. Unlike the liver, the pancreas and the blood vessels around it are retroperitoneal organs with little deformation or displacement in general surgeries. In our study, we developed an intraoperative navigation system for laparoscopic pancreatic surgery. We are the first to realize intraoperative navigation by fusing the preoperative 3D virtual model with the real-time laparoscopic images in pancreatic surgery. Considering the flexible change in the surgical position in laparoscopic pancreatic surgery, the intraoperative navigation function can be achieved manually or automatically using an optical orientation and registration system. To better meet the operator’s demand, the operator can build a 3D virtual model consisting of all the structures that he/she is interested in by selecting the area of interest using the CT image in the navigation system. We tested the intraoperative system with laparoscopic simulators and pilot clinical cases and it worked well.

In the preclinical tests with rigid and phantom models, the covered structures were clearly and correctly displayed on the fusion image. And the practicality of our navigation system was proved by the NASA-TLX workload measurement. After achieving satisfying results in preclinical tests, we tried the navigation system in pilot cases. In the pilot cases, the 3D model was fused manually to show the tumor and the blood vessels in the deep tissue. The results indicated the possibility that the operator with limited experience of laparoscopic pancreatic surgery could use the navigation system to have a comprehensive visual field and complete the procedure as safely and efficiently as that performed by an experienced operator.

Clinical tests involving more patients are required to further evaluate the actual clinical outcomes of our intraoperative navigation system. First, clinical studies with the fusion function of automatic optic orientation are required to evaluate the accuracy of registration. Second, comparative studies of laparoscopic pancreatic surgeries are required to explore the actual clinical outcomes of the intraoperative navigation system, such as a reduction in the operation time, and intraoperative blood loss and other clinical outcomes.

## Conclusions

Our study showed that the operators could clearly observed the anatomical structures in the deep tissue using our intraoperative navigation system, which has the potentiality of helping achieving a more safe and efficient laparoscopic pancreatic surgery. Also, it was easy to use. Formal clinical studies involving more patients will be conducted to further prove the clinical practicality of the intraoperative navigation system applied in laparoscopic pancreatic surgery.

## Data Availability

The datasets used and/or analysed during the current study available from the corresponding author on reasonable request.

## Abbreviations

3D: Three dimensional
CT: Computed tomography
DICOM: Digital imaging and communications in medicine
NASA-TLX: National aeronautics and space administration-task load index
